# The Effect of Size of Materials Formed or Implanted In Vivo on the Macrophage Response and the Resultant Influence on Clinical Outcome

**DOI:** 10.3390/ma14164572

**Published:** 2021-08-14

**Authors:** Dale Feldman

**Affiliations:** Department of Biomedical Engineering, University of Alabama at Birmingham, Birmingham, AL 35294, USA; dfeldman@uab.edu; Tel.: +1-205-807-2445

**Keywords:** chronic inflammation, host response to particulates and fibers, macrophage activation

## Abstract

Both the chemistry and size of a material formed in vivo, or an implanted biomaterial, can alter the in vivo host response. Within the size range covered within this review, over 1 μm, chemistry is only important if the solid material is unstable and leeching small molecules. The macrophage activity and the resultant inflammatory response, however, are related to the size of the solid material. The premise of this review is that differences in size of the solid material, in different cases, can be the reason why there is some individual-to-individual variation in response. Specifically, the inflammatory response is enhanced when the size is between 1–50 μm. This will be looked at for three configurations: spherical particulate (silicone oil or gel from breast implants), elongated particulate (monosodium urate [MSU] crystals in gout or in kidney stones), and fibers (e.g., polyester used in fabric implants). These specific examples were selected because many still believe that the clinical outcome for each is controlled by the surface chemistry, when in fact it is the size. In each case, specific studies will be highlighted to either show a mechanism for creating different sizes and therefore a differential biological response (first three) or how changing the size and shape (diameter and spacing of fibers, in this example) can affect the response and can help explain the different responses to fabric implants found in vivo within the 1–50 μm size range. It was found that polyester fibers under 70 μm had a significant increase in macrophage response. Further, it was found that compounds found in synovial fluid could limit MSU crystal size. In addition, it was shown that plasma with low triglyceride levels emulsifies silicone oils to a greater extent than plasma with higher triglyceride levels. Therefore, in three cases it appears that differences in the inflammatory response between individuals and between different implants could be explained just by the size of the material formed or implanted.

## 1. Introduction

Both the chemistry and size of a material formed in vivo, or an implanted biomaterial, can alter the in vivo host response [1,2,3,4,5,6,7,8,9,10,11,12,13,14,15,16,17,18,19,20,21,22,23]. For an implanted biomaterial it can be part of the implant, pieces broken off, or chemicals leached out. It can be a positive adaptive response or one leading to pathology (inflammation, immune response, cancer, toxicity, etc.) [15]. In tissue, typically it is small molecules or ions released that lead to implant pathology [1,7,15]. These released chemicals can trigger cell adaptive responses directly or indirectly by binding to other compounds, through biotransformation or by causing mutations [1,7,15]. There are two separate bioprocesses that will be examined here: (1) response to the introduction of a solid material and (2) production of solid materials from chemicals found in the body or from the implanted material.

When a stable solid object is encountered, the size and shape tend to control the host response [1,2,3,4,5,6,15]. These objects can be implanted as part of a medical device or form in vivo from biological materials. The size and shape of the object affects the macrophage response. Changes in the macrophage response can alter the inflammatory response and immune response [1,2,3,4,5,15].

There are three main examples, however, where chemistry for a stable material still matters. One is when it is in contact with blood, one is if the object contains foreign protein, and the third is for nanoparticles. The surface chemistry can affect the sequence and the way blood proteins attach to the material, which can have a significant effect on the blood clotting cascade within minutes of contact [4,15]. In tissues, however, the host response develops over hours and days, and by that time the surface of the material will not be much different even with different surface chemistries [4,15].

The immune response can be triggered by foreign proteins, and therefore surface chemistry can determine whether it is treated as an antigen [4,7,15,20]. In the nanometer range, it appears particles begin to act more like chemicals (with proteins typically around 10 nm) and surface chemistry can be very important [16]. Biologic materials and polyethylene glycol attached to the nanoparticles can alter the uptake of the nanoparticles, particularly between the M1 and M2 phenotypes [8,16].

Both porosity and surface texture of a biomaterial have been shown to affect the ECM (extracellular matrix) production altering the fibrous capsule and presence of dead space, which can cause implant pathology [1,2,3,4,5,6,7,15]. The size alone has been shown to increase activation of the macrophages [1,2,3,4,5,6,7,15]. This has been shown in relation to the size of particulates formed from friction and wear after joint replacement [1,2,3]. Activation typically occurs when the smallest dimension is under 50 μm, and macrophages try to phagocytize the material [1,2,3,4,5,6,7,15]. There also appears to be at least two changes when the size goes below 1 μm [1,2,3]. Particles under 1 μm seem to be easily phagocytized and cleared, reducing the inflammatory reaction [1,2,3]. However, again they can also have surface treatments that can affect the uptake of the particles in the nanometer range.

This review will concentrate on the 1–50 μm size range, where activated macrophages can cause both local and systemic effects by inducing an inflammatory response [1,2,3,4,5,6,7,15]. This inflammation can enhance the immune response, slow healing, cause tissue necrosis, increase the infection rate, and facilitate carcinogenesis [4,15].

Recently, two phenotypes of macrophages have been identified (M1 and M2) [8]. M2 is the more common type, and when activated produce growth factors to aid in wound healing (stimulate fibroblasts to produce collagen as well as enhance angiogenesis) [8]. M1 macrophages, when activated, produce pro-inflammatory cytokines and are responsible for cleaning up a wound via the inflammatory response [8]. They also can present antigens to lymphocytes to stimulate an immune response [4,15].

Although M2 is the normal state of a macrophage, they can be transformed into the inflammatory (M1) phenotype [8]. It is likely that the presence of a foreign material triggers this transformation, which ends up with a chronic inflammatory response and fibrous encapsulation [8,15]. There, however, appears to be a size range that increases the activity of these M1 macrophages [1,2,3,4,5,6,7,8,15]. It is possibly the same trigger as the one that causes macrophages to combine to form giant cells: the foreign material is small enough to be surrounded by or phagocytized by a macrophage, but cannot be broken down enough to be taken to the lymphatic system to be removed from the body [15]. Again, there appears also to be a lower limit to the size where the macrophage can easily remove the foreign material without a significant change in activity [15,22].

The reason for the two limits are not known for sure. They are probably related to the ease of removal of the foreign material, and either the speed or energy requirements to do so (or both) [15]. At the upper limit (50 μm) the macrophage can first start to surround the foreign material in an attempt to remove it [1,2,3,4,5,6,15]. At the lower limit, the macrophage can quickly and/or easily remove the foreign material [15,16,17,18,19,20,21,22]. The timing may also be what triggers the fibrous encapsulation response. If it cannot be broken down and/or removed quick enough, the inflammatory response leads to walling off of the foreign material [4,15].

The main premise of this review is that differences in size of the solid material, in different cases, can be the reason why there is some individual-to-individual variation in response. Specifically, the macrophage response leading to inflammation is enhanced when the size is between 1–50 μm. This will be looked at for three configurations: spherical particulate (silicone oil or gel from breast implants), elongated particulate (monosodium urate [MSU] crystals in gout or in kidney stones), and fibers (e.g., polyester used in fabric implants). These specific examples were selected because many still believe that the clinical outcome for each is controlled by the surface chemistry, when in fact it is the size [1,2,3,4,5,6,7,19,22,23].

In each case, specific studies will be highlighted to either show a mechanism for creating different sizes and therefore a differential biological response (first three) or how changing the size and shape (diameter and spacing of fibers, in this example) can affect the response and can help explain the different responses to fabric implants found in vivo within the 1–50 μm size range. Additionally, these studies can help when designing devices or treatments to produce a more desirable (more biocompatible) clinical outcome: the concept of “safety by design” by preventing solid materials in the 1–50 μm size range [1,2,3,4,5,6,7,14,16].

## 2. Gout and Kidney Stones

Crystallization of monosodium urate (MSU) is the cause of gout as well as about 10% of kidney stones [24,25,26,27,28,29]. Gout is the most common inflammatory arthritis affecting about 1% of the population (5% of arthritis patients) [24,25,26]. Kidney stones occur in about 10%–14% of the population (about 1% with MSU crystals) [25,26,27,28,29].

### 2.1. Role of Size and Shape

In both cases, size and shape of the crystals affects the clinical presentation. For kidney stones, this determines residence time in the various parts of the body from the kidney to excretion through the urinary system. For gout, it is likely that the size (mostly diameter) of MSU crystals is what leads to macrophage activation and the inflammatory response [1,2,15].

A pre-requisite for gout is excessive blood levels of soluble urate, one of the final products of the metabolic breakdown of purine nucleotides [30]. Hyperuricemia is typically defined as occurring above the saturation point of MSU, at serum urate levels > 6.8 mg/dL [31].

Although the exact mechanism for gout is not known, it is generally accepted that interaction of MSU crystals with leucocytes leads to local inflammation and the production of chemicals that increase the amount of MSU crystals, amplifying the clinical signs of gout: swelling, redness, and pain [24,26]. It is still, however, not agreed upon what causes MSU crystallization as well as how the MSU crystals trigger the inflammatory response [1,7,22].

There is some belief that the surface chemistry is important, typically from an immunological perspective [32,33,34]. It has been shown that isolated MSU crystals are typically coated with immunoglobulins [35,36]. The surface concentration of immunoglobulins decreases as inflammation resolves, while the apolipoprotein Β surface concentration increases [35,36]. Further, the cationic F_ab_ portion of the antibodies bind to urate with the F_c_ portions exposed [37]. The F_c_ portions then may play a role both in the ability of the crystal to activate complement as well as the ability of F_c_-receptor-bearing cells to phagocytose crystals and undergo cell activation [35,38].

It is, however, also suggested that it is the activated resident tissue macrophages, which secrete inflammatory cytokines including IL-1β [39,40], leading to complement activation and infiltration of neutrophils, with production of additional pro-inflammatory mediators such as PGE_2_ and LTB_4_ [32].

### 2.2. Treatment

#### 2.2.1. Prevention

Not knowing the exact mechanisms makes it difficult to select appropriate strategies or even understand how they work. Prevention strategies either try to control the crystallization phase or limit the inflammatory phase, with current drug therapy mostly related to the inflammatory phase [25,41]. All current prevention drugs are aimed at reducing the uric acid concentration by increasing excretion or reducing production [25,41,42]. In some cases, this can actually trigger an attack (possibly by creating more nucleation sites) [43]. The inflammation, in these cases, is reduced by preventing or limiting the amount of crystals formed [25,44,45].

#### 2.2.2. Treating a Gout Attack

Strategies include reducing the serum uric acid level, suppressing the inflammatory response, or trying to dissolve the crystals. The anti-inflammatory drugs normally reduce the production of inflammatory compounds such as prostaglandins [25,41] or can also be used to reduce phagocytosis [25,41]. Additionally, uric acid kidney stones have been successfully dissolved, in 70–80% of the cases, by lowering the pH with citrate [46]. Another strategy, based on the immune response, is to prevent or reduce complement activation [30,38,39,40].

#### 2.2.3. Treatment Based on Size

**Figure 1:**
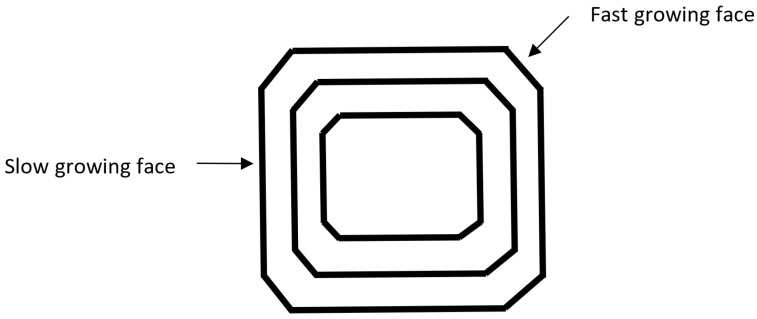
Relative growth rates as a crystal grows.

A prevention strategy based on size, since studies have shown that keeping MSU crystals under 0.5 μm can prevent a gout attack [34], has not been fully explored. An approach can come from the fact that only 2% to 36% of hyperuricemic (above saturation levels of uric acid in their serum) patients, with approximately 5–10 years of follow-up, develop gout [25,47,48]. This implies that there are chemicals (as well as environmental factors such as pH) found in the body that can limit MSU crystallization size or amount above the threshold [47]. Therefore, instead of reducing uric acid concentration or increasing its solubility, crystallization could be limited by reducing the nucleation rate or affecting growth rate of the crystals [25,45]. Limiting crystal size can be done by slowing the growth rate preferentially on the fastest growing faces (Figure 1).

### 2.3. Developing a Treatment Based on Size

#### 2.3.1. Methodology

The MSU crystals were made using a modification of Seegmiller’s method [44]. In this technique, 1.006 gm of reagent grade uric acid (J.T. Baker Co., Phillipsburg, NJ, USA) was dissolved in 194 mL of boiling water with 6 mL of 1 N NaOH. The pH was adjusted to 7.4 (physiological pH) by adding additional NaOH. The solution was filtered and a known amount of the chemical being tested was added after the first filtration and before the solution was allowed to stand for nine hours and then dried in an oven at 60 °C. Each chemical additive was tested at a variety of concentrations up until saturation (Table 1). The crystals formed were analyzed using a light microscope with polarizing light to check for the negative birefringence characteristic of MSU. X-ray diffraction (XRD) was performed to check for changes in crystal structure. SEM analysis was done to observe changes in morphology and make size measurements. Changes in color were also noted (indicative of adsorption on the crystal surface).

#### 2.3.2. Results

The model was not intended to mimic in vivo conditions or accurately predict the change in size expected in gout patients. It did, however, isolate chemicals that have the potential to limit MSU crystallization (either amount or size). Further, it also gave an indication of the mechanism involved in any alteration in the crystallization process. There was a clear difference between the third of the compounds that had an effect vs. those that did not. It appeared that riboflavin, niacin, calcium (as CaSO_4_ and Ca_3_(PO_4_)_2_), methylene blue, and fuchsin could reduce MSU crystal size in a dose dependent manner. Pyridoxine HCL, β-carotene, lysozyme, and xanthine appeared to reduce crystal size, but only one concentration (plus the control) was used; therefore, a dose dependent response could not be shown. Additionally, all but pyridoxine HCL and β-carotene reduced the amount of precipitate formed.

#### 2.3.3. Ramifications

There seemed to be at least five mechanisms (with some overlap) by which these compounds could limit crystal size: adsorption, syn-crystallization (more than one crystal growing together), ion incorporation into the crystal, a modification of the solubility of uric acid, or degradation of the crystal. Adsorption and syn-crystallization can be shown by a change in the color of the crystals. Changes in XRD relative peak intensity indicates either syn-crystallization or ion incorporation (which can also lead to syn-crystallization), with the exact change able to help distinguish between the two. Changes in amount of precipitate and/or changes in pH are indicative of changes in solubility of uric acid. Degradation of the crystal can be seen by changes in shape and smoothness of the crystals.

Coating of the growing crystal with adsorbed compounds or a different crystal (syn-crystallization) can alter shape and/or limit size by inhibiting growth on one or more crystal surfaces. Figure 1 shows the effect of having different growth rates on different faces, which can also result in a color change. Incorporation of ions into the growing crystal can change the relative growth rates of crystal surfaces and therefore effect shape and size of the final crystal, potentially through syn-crystallization [49].

The most effective compounds appeared to be ones that are incorporated into the growing crystal via syn-crystallization or competing with sodium for changes in the crystal chemistry. It is expected that compounds with a similar pyrimidine structure to MSU more easily exhibit syn-crystallization. This appears to be true for riboflavin, xanthine, pyridoxine HCL, and methylene blue.

The study showed the feasibility of specific compounds found in the body to limit the size of MSU crystals in vivo. This could also be used as a prevention medication, if levels are too low in joint synovial fluid. Although there is a lot to do to develop a treatment, this study shows how crystal size can be part of the reason why only a fraction of hyperuricemic individuals develop gout.

## 3. Silicone Breast Implants

There are about 300,000 breast implantations per year for either cosmetic augmentation or reconstruction following mastectomy [50,51]. The current implants are silicone rubber bags filled with saline or silicone gel. The types of implants and options have changed over the years.

The first recorded attempts at altering the shape of human breasts were conducted in the 20th century using wax, fat grafts, and other tissue grafts [51]. After World War II, a polyether (etheron) was used. None of these were very successful [50,51].

In the late 1940s through the early 1960s, silicone oils were often directly injected into breast tissue [51]. The effect, in this case, was short lived due to systemic distribution of the oil. To combat this, various irritant additives such as the “sakurai formula” were formulated [52]. The “irritant” was used to stimulate a fibrous capsule around the injection to keep it in place and reduce systemic distribution of the silicone [52]. The lack of standardization of the “irritant” led to undesirable host responses, from severe foreign body responses to even cancer [19]. This led to the first ban on silicone by the FDA, in 1965, on silicone injections for breast augmentation [19].

The first silicone gel breast implants were developed in 1963 [53]. The silicone gel was placed in a silicone elastomer bag (polydimethylsiloxane—PDMS) to prevent free gel in the tissue, avoiding both the biocompatibility concerns and the loss of cosmetic function over time [53].

The design of these silicone gel implants has changed over the years. The original implants (1960s–1970s) had a relatively thick shell (~0.04 cm) and a relatively viscous gel [54,55]. Many of the implants became hard as a result of capsular contraction of the fibrous capsule around the implant [55].

Since the hardness of the breast was originally ascribed to the thickness of the implant shells, breast implants with thinner shells (~0.08 inches), and less viscous (“more responsive”) gels were introduced in the 1970s [56]. These second generation implants, however, resulted in an increase in the amount of gel components found in the surrounding tissue compared to first generation implants, due to implant rupture as well as diffusion through the shell (gel bleed) [57,58].

The third generation implants, in the 1980s, were designed to reduce the amount of gel in the tissue by using an intermediate thickness shell and often had a barrier layer (typically a fluorosilicone) [59]. Another design change was making the surface porous in an attempt to reduce capsular contraction [19].

Because of uncertain risks, however, the FDA banned silicone breast implants in the 1990’s for all indications except for augmentation after mastectomy [19,60]. Later, the FDA re-approved (in 2006) gel filled implants based on studies from mastectomy reconstructions, but required additional long-term post market surveillance studies [19,50].

### 3.1. Current Complications

Almost half of women with breast augmentation surgery have had complications including pain, capsular contraction, infection or the need for additional surgery [19,50,61,62,63,64,65]. About 25% of the women with silicone breast implants have had to have them removed vs. only 8% for saline filled [19,50]. The average lifespan is between 7 to 12 years [19,50].

The actual acceptable rates necessary for the re-approval were:

1. 20% capsular contraction, 20% asymmetry, 13% wrinkling, and 10% visible implants after 5 years [19,50].
2. 15% rupture rate after 8 years and a 50% reoperation rate within 10 years [19,50].

#### 3.1.1. Cause

The difference in success rate (need for removal) is significantly different between saline filled and gel-filled. [19,50]. The main difference between the two is the presence of gel outside the implant, which can lead to inflammation, which can lead to fibrous encapsulation, which can lead to pain and/or rupture necessitating removal of the implant. The gel can come out from gel bleed or implant rupture [19,50].

##### Gel Bleed

Although the gel-in-shell strategy was used to overcome the FDA ban on direct injection of silicone, uncross-linked PDMS molecules in the gel still tended to diffuse across the highly cross-linked silicone shell and into the physiological environment [19,66,67]. The extent of bleed is dependent on the gel composition (both molecular weight and amount of cross-linking), the shell thickness, and the surface area to volume ratio of the prosthesis [19,66,67].

The composition of gel bleed has been shown to include all the uncross-linked PDMS fluids with a molecular weight of 158 K or less [67,68,69,70,71,72]. The diffusion through the shell is lower as the molecular weight increases [67,71]. The bleed composition has an average molecular weight of 9000–24,000 [67]. The amount and molecular weight of gel bleed varies between the three shell thicknesses as well as with the use of a barrier layer [67,68]. Further, the relative non-uniformity in gel and shell composition across different manufacturers, and even across different batches of a single manufacturer (plus the three different generations) makes it difficult to obtain a precise estimate [67,68,72].

Bleed rate has been estimated at 200 mg/yr for a 230 c implant or about 0.6% per year, but rates as low as 60–100 mg/yr to as high as 2.1 gm/yr have been reported [19,72,73]. The role of the barrier layer is controversial, with some believing it not only alters the composition of the bleed but also decreases the amount of bleed by 90% [67]. Others, however, suggest that the barrier layer only serves to delay the onset of the bleed and it reaches the level of the other implants once the gel saturates the shell, after two to three years [74,75].

##### Implant Rupture

When the shell ruptures, a much larger quantity of gel/oil is released into the environment than that with gel bleed alone, and immediate ex-plantation is recommended [76]. The causes of this failure are still under debate. Although the thickness of the shell has an effect on the time to failure, the 7–12 year average lifetime is for the current “intermediate thickness” shells [19,50].

Since, it has been shown that the force to break the shell decreases over time; this could be the main cause of the limited lifetime [77,78,79,80,81,82]. Some have linked this change in properties to the gel diffusing into the shell, which it does before the gel is released (gel-bleed) [19]. In the early stages of implantation, once the shell becomes saturated with gel, it swells and becomes about 30% weaker [81,82,83,84]. For current implants, 3 lbs is sufficient to break the shell after five years, compared to 4–5 lbs new [81]. The stress to failure decreases by 17–20% in 1–2 months, 32–34% in 1 year, 40% in 6–12 years, and about 50% in 9–10 years [81]. The strain to failure reduces from 1000% to 300% by 8 years [81].

#### 3.1.2. Differential Response Due to Size

Again, with the likely reason why the complication rate is higher for gel-filled versus saline filled being the presence of gel bleed, it is unclear why the response can be significantly different between patients even without the silicone shell breaking [19]. One theory is that there is a difference in the size of silicone oil droplets after they leach out of the implant [1,2,3,19].

The general response to silicone oil is similar to other particulates: a chronic inflammatory response with a severity dependent on the size, amount, and duration of exposure [85,86,87]. Again, similar to other particulates, the most macrophage activity occurs when droplets are below 50 μm in size [1,2,3,19,88]. The length of time the macrophages stay active, producing cytokines, is however controversial and requires further study. Some studies suggest it is at least in the order of months [7,89].

As anticipated, since the foreign body response is dependent on the amount of silicone oil present, certain levels of silicone oil appear to be well tolerated. For example, small dosages (3.5–55 mL total with less than 0.07 cc/time) of Dow Corning 360 medical grade silicone (MDX4-4011 (350 cSt)) delivered in multiple dosages every one to two weeks, with massage, becomes dispersed in tissue planes and leads to only a mild response, which is resolved within six months [7,90,91,92]. It appears that breast tissue requires a lower amount of silicone to elicit a response than does other tissue [7,91,92].

In this case, it appears stable silicone oil emulsion is formed in the tissue, with droplets ranging from submicron to 200 μm, with means of 10–15 μm, typically seen [7,19,91,92]. There is also evidence in the ophthalmological literature that silicone oils, used to replace vitreous humor after retinal reattachment surgery, can emulsify. For these ocular applications, silicone oils > 5000 cSt viscosities are recommended to prevent emulsification [93]. Surfactants are needed to create a stable emulsion, with phospholipids serving this function in vivo and thus influencing the extent of emulsification [94,95,96]. It has been hypothesized that differences in plasma chemistry may determine the extent of emulsification that in turn would lead to the differential biological responses observed clinically [19].

### 3.2. Methods

Silicone oil of the type found in gel bleeds was mixed with two plasma specimens. One had low (90 mg/mL) triglyceride levels and one had higher (284 mg/mL) triglyceride levels. The amount of emulsification was characterized.

### 3.3. Ramifications

The plasma with normal (90 mg/mL) triglyceride levels emulsified the silicone oils to a greater extent (up to 100% more) than plasma from persons with high (284 mg/mL) triglyceride levels [97]. Although the difference in droplet size and number of droplets as well as the fate of the droplets and their effect on the inflammatory response is unknown for a given patient, the study showed a possible mechanism to help explain the variation in clinical response to silicone gel. In this case, the volume of droplets was altered by a change in triglyceride level in the plasma. It is likely that both size and volume affect the inflammatory response. Therefore, a composition of blood test or even an emulsion test could be used to determine susceptibility to capsular contraction and could possibly lead to a treatment. There would be many studies required before a clinical intervention could be used. Again, the goal is merely to show a possible reason why size can alter the biological response to breast implants.

## 4. Fibrous Implants

For implants that have a fibrous component there are many variables that can affect the macrophage response and therefore clinical outcomes. Assuming the fibers are smooth, non-degradable, and not leaching any small molecules from the bulk or surface, the chemical make-up does not make a significant difference in macrophage response [15]. Various configuration modifications can alter the macrophage response, including surface roughness, fiber diameter, and fiber 3D orientation [5,15,98,99,100,101,102,103,104].

In some cases, the mechanical properties of the 3D fiber structure (fabric) are also critical for clinical success. One example is hernia meshes where the strength and stiffness of the tissue/fabric composite and the attachment of the fabric to surrounding tissue are important for a successful clinical outcome [5,104,105,106]. In these cases, the inflammatory response triggered by the macrophages can lead to inadequate mechanical properties [15,23,107,108,109,110,111,112].

### 4.1. Potential Size and Shape Effects

For soft tissue implants, the porosity can have a significant effect on the macrophage response and therefore amount and type of tissue ingrowth [15]. The effect of altering the porosity (pore size, pore shape, amount of interconnectivity, and percent porosity) on tissue ingrowth has been extensively explored [15,98,99,100,101,102,106].

Different minimum pore sizes have been established for blood vessel ingrowth (at least 40 μm) [100]. Further, pores that are too large can reduce the ability of the implant to serve as a scaffold [15]. For fabric implants, the average distance between fibers in 3D has been used for pore size [15,100]. To optimize the scaffolding effect, a pore size of 100 μm has been suggested for collagen based artificial skin [98,99], and about 75 μm for hernia meshes [106].

Failure of some porous medical devices are directly linked to the inflammatory response. Examples include: the porous polyurethane coatings on breast implants, which had an excessive inflammatory response [19], meshes to support the uterus or bladder creating an inflammatory response, which would eat through the vaginal wall, and hernia meshes that do not integrate well with the surrounding tissue [15,19,105,106,107,108,109,110,111,112].

The link between size and inflammation for fabric implants is the size of the fibers. For a macrophage, the fiber diameter is important, since the length is too long to be engulfed [15]. The inflammatory response can influence the clinical outcome in many ways. If individual fibers are surrounded by giant cells, they reduce the useful pore size, in some cases below the 40 μm size needed for blood vessel ingrowth [100]. The inflammatory response will not subside if the fibers cannot be broken down, and a thicker fibrous capsule is formed around the fabric [5,100,103,104]. In addition, little healing occurs during the inflammatory phase [100]. There also is the constant release of inflammatory cytokines, which can damage surrounding tissue [15].

Fibers are different than the previous examples, in that even if a macrophage can surround the diameter of the fiber, it is much longer than the macrophage (or giant cell formed), which forms a sleeve around it. This presents a different scenario, in which the macrophage can be activated, but cannot remove or isolate the foreign material. Although this can happen in the previous cases, crystals too long or droplets too large to be phagocytized, a fiber in a fabric could have multiple macrophages forming short sleeves along the whole length.

Asbestos fibers are possibly close to the transition between the previous examples (MSU crystals and silicone droplets) and fibers. The asbestos fibers typically are 5 μm or less in length (but can go up to 40 μm) and 3 μm or less in diameter [113]. Both in vitro toxicity and in vivo studies seem to indicate that fibers need to be at least 4 μm long (with an increase of 8 μm over 4 μm) to elicit a significant response [113]. The distinction between short and long fibers is typically 5 μm. The role of diameter is unclear in this size range, and may affect retention more than activation [114].

The asbestosis fibers can cause inflammation, which leads to fibrosis, which can lead to cancer, most likely due to cancer cells inside a fibrosis capsule being protected from the immune system long enough to create a critical mass [15,113]. For asbestosis, the size and shape also can determine the residence time in the lungs. To cause a fibrotic response, the fibers have to be trapped in the lungs, which is more likely with the longer thinner fibers (which is why sometimes the length to diameter ratio is cited) [15,113]. Short fibers are also easily phagocytized and removed, eliciting a short-term inflammatory response that is quickly resolved [113].

It is possible that the length of these fibers has most of its effect due to entrapment in the lungs, for asbestosis. This is probably why long fibers are not removed by phagocytosis as easily as short fibers (or crystals and droplets). There is probably a transition, in length, closer to the size of macrophages and giant cells where phagocytosis is no longer possible [15].

Although there are still some that claim the chemical makeup of asbestosis is the main culprit, the size seems to explain the differential response to different fibers. Again, normally small molecules have to leach out for chemistry to have a significant effect [15].

It is also possible that long fibers that cannot be encapsulated do not have a lower limit on diameter, since they cannot be phagocytized and removed. However, it is expected that the diameters under 50 μm would lead to more macrophage activation, as they do for particles and droplets [15]. Most sutures are above this size until 7–0, although all sizes of monofilament sutures seem to have at most a short lived inflammatory response [104]. In a number of fabric implants, however, typically those with polyethylene terephthalate (dacron), the fibers are under 50 μm [100].

Since dacron fibers are stable in vivo, the chemistry most likely does not play a big part, although some can leech chemicals used in processing in the short term [1,5,15,103]. There have been many studies of individual fibers of different diameters, but few with fabric implants. The individual fibers were sutures above the 50 μm size or between 2 μm and 40 μm [5,103,104]. Although the transition above and below 50 μm was not studied, there did seem to be a reduction in response below 6 μm [5,103,104]. In a study with a dacron velour implant with fibers 25–40 μm in diameter, a long-term chronic inflammatory response with fibrous encapsulation was seen [100].

A study was conducted [23] to determine if the fiber diameter threshold was similar to the particle threshold of 50 μm for a fiber mesh. In this study, the meshes had varying fiber spacings as well as fiber diameters. 

### 4.2. Methods

**Figure 2:**
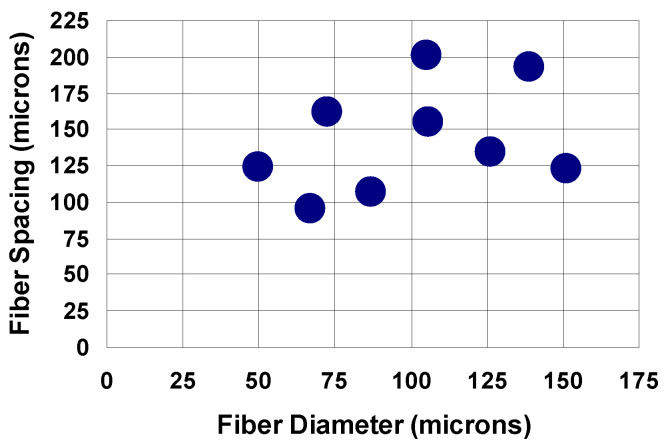
The measured fiber diameter and fiber spacings.

The fabrics used in this study [23] were made of non-medical grade monofilament polyethylene terephthalate (PET) fibers in a plain square weave (Tetko, Inc., Briarcliff Manor, NY, USA). The fiber diameters and spacings of the fabrics are shown in Figure 2.

Each fabric piece was cleaned, sterilized and implanted in a rabbit model. Up to six pockets were made on each side of the spine to accommodate up to 12 specimens per animal. Nine fabrics were studied at two weeks (n = 6) and six fabrics at four weeks (n = 4). Histomorphic analysis was conducted on cross sections to determine the tissue response.

### 4.3. Results and Discussion

**Figure 3:**
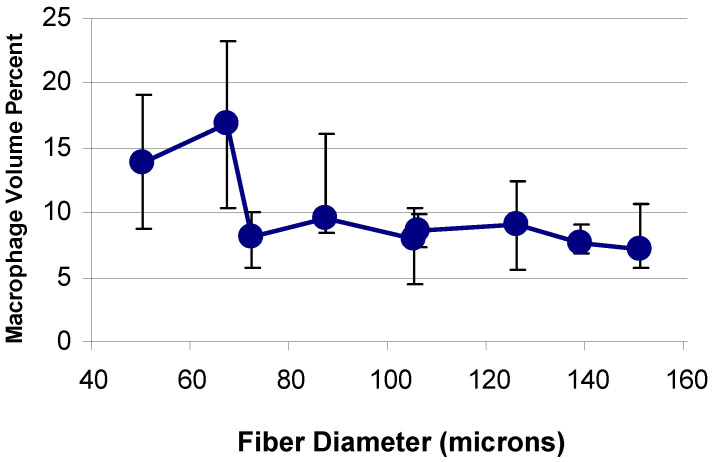
The macrophage response versus fiber diameter for the 2-week study.

The increase in macrophage response occurred between 72 μm and 67 μm fiber diameters at both two and four weeks post implantation (Figure 3) [23]. Although the giant cell response increased as the fiber diameter decreased, the threshold was not as dramatic and seemed to occur at a slightly higher fiber diameter, than for the macrophage response [23]. Additionally, fabrics with fiber diameters below 70 μm showed an increase in inflammation and decrease in tissue repair [23]. The fiber spacing showed some differences, but nothing significant within the ranges used in this study [23].

The difference in macrophage response, inflammation, and tissue repair seen above and below the threshold value (about 70 μm) show the importance of fiber diameter when used in implantable devices [23]. This can help explain why certain meshes do not serve as good scaffolds and therefore can lead to mechanical failure in hernia repair or erosion of surrounding tissue when used for slings to support the bladder or uterus.

## 5. Conclusions

Therefore, in three cases it appears that differences in inflammatory response and resultant clinical outcomes between individuals and between different implants could be explained by the size of the material. These represent three different shapes (spherical, needle shaped, and long fiber). In each case, some have blamed the pathology on the chemistry [15]. However, when a material is stable or the breakdown products are relatively inert (i.e., silicone or uric acid) the size tends to control the inflammatory response [15]. There are other cases where size is important, including asbestosis and wear debris in artificial joints [15]. Asbestosis is helped by the shape, allowing the silicate fibers to become lodged in the lungs as well as is the longest word in the English language currently: pneumonultramicroscopicsilicovolcanoconiosis.

Two of the examples are materials that form in vivo (silicone oil droplets and MSU crystals) and plasma chemistry can help determine the size of the materials and therefore control the pathology. This can both be used to explain the differential response from individual-to-individual, as well as suggest preventative measures. The third example (polyester fibers) shows the importance of fiber diameter in implant pathology related to the inflammatory response.

## Figures and Tables

**Table 1:** Chemical additives used to effect MSU crystals.

Vitamins and Minerals	Found in Foods
β-carotene	glucose
thiamine (B_1_)	caffeine
L(+) ascorbic acid	citric acid
riboflavin (B_2_)	starch
pyridoxine HCL (B_6_)	glycine
niacin (B_3_)	monosodium glutamate
vitamin pill	β lactose
calcium	nicotinamide
	pyruvic acid
**Associated with Gout**	**Also Found in the Body**
Ca_3_(PO_4_)_2_	potassium chloride
CaSO_4_	adenine
ethyl alcohol	L-leucine
dextran	liver residue
fuchsin	pancretin
lysozyme	pepsin
brilliant green	trypsin
DL lactic acid	urea
xanthine	penicillen
methylene blue	

## Data Availability

Data sharing is not applicable to this article.

## References

[1] Shanbhag A., Jacobs J., Black J., Galante J., Glant T. (1994). Macrophage/particle interaction: Effect of size, composition, and surface area. J. Biomed. Mater. Res..

[2] Gonzalez O., Smith R., Goodman S. (1996). Effect of size, concentration, surface area, and volume of polymethylmethacrylate particles on human macrophages in vitro. J. Biomed. Mater. Res..

[3] Nagse M., Wise D. (1995). Host reactions to particulate biomaterials. Encyclopedic Handbook of Biomaterials and Bioengineering.

[4] Black J. (1992). Biological Performance of Materials: Fundamentals of Biocompatibility.

[5] Sanders J., Bale S., Neumann T. (2002). Tissue response to micro-fibers of difference polymers: Polyester, polyethylene, polylactic acid, and polyurethane. J. Biomed. Mater. Res..

[6] Mooney D., Langer S., Bronzino J. (1995). Engineering Biomaterials for Tissue Engineering: The 10–100 Micron Size Scale. The Biomedical Engineering Handbook.

[7] Naim J.O., van Oss C.J., Ippolito K.M.L., Zhang J.-W., Jin L.-P., Fortuna R., Buehner N.A. (1998). In vitro activation of human monocytes by silicones. Coll. Surfaces B Biointerfaces.

[8] Kun Yeong L. (2019). M1 and M2 polarization of macrophages: A mini-review. Med. Biol. Sci. Eng..

[9] Palsson B., Bhatia S.N. (2004). Tissue Dynamics. Tissue Engineering.

[10] Goss R., Cohen I., Diegelmann R., Lindblad W. (1992). Regeneration Versus Repair. Wound Healing: Biochemical and Clinical Aspects.

[11] Palsson B., Bhatia S.N. (2004). Cellular-Fate Processes. Tissue Engineering.

[12] Goss R., Cohen I., Diegelmann R., Lindblad W. (1992). Tissue Repair in the Mammalian Fetus. Wound Healing: Biochemical and Clinical Aspects.

[13] Palsson B., Bhatia S.N. (2004). Coordination of Cellular-Fate Processes. Tissue Engineering.

[14] Fries R.C. (2005). Reliable Design of Medical Devices.

[15] Feldman D. (2019). Quantification and modeling of biological processes for tissue engineering and regenerative medicine. Biomed. J. Sci. Tech. Res..

[16] Qie Y., Yuan H., von Roemeling C.A., Chen Y., Liu X., Shih K.D., Knight J.A., Tun J.A., Wharen R.E., Jiang W. Surface Modification of Nanoparticles Enables selective Evasion of Phagocytic Clearance by Distinct Macrophage Phenotypes. www.nature.com/scientificreports/6:26269.

[17] Feldman D., Czuwala P., Kelpke S., Pandit A., Wilson D., Wise D. (1995). A biocompatibility hierarchy: Justification for biomaterial enhanced regeneration. Encyclopedic Handbook of Biomaterials and Bioengineering.

[18] Feldman D.S. (2019). Biomaterial enhanced regeneration design research for skin and load bearing applications. J. Funct. Biomater..

[19] Kilpadi D., Feldman D., Wise D. (2000). Biocompatibility of silicone gel breast implants. Biomaterials Engineering and Devices: Human Applications.

[20] Granchi D., Cavedagna D., Ciapetti G., Stea S., Schiavon P., Giuliani R., Pizzoferrato A. (1995). Silicone breast implants: The role of immune system on capsular contracture formation. J. Biomed. Mater. Res..

[21] Feldman D.S., McCauley J.F. (2018). Mesenchymal stem cells and transforming growth factor-β3 (TGF-β3) to enhance the regenerative ability of an albumin scaffold in full thickness wound healing. J. Funct. Biomater..

[22] Feldman D., Myerson A. (2017). Feasibility of a strategy to prevent gouty arthritis through limiting crystallization of monosodium urate. Int. J. Drug Res. Tech..

[23] Feldman D., Ferguson D. (2017). The effect of fiber spacing and fiber diameter on soft tissue ingrowth for polyethylene terephthalate. J. Biomed. Imag. Bioeng..

[24] Roddy E., Doherty M. (2010). Epidemiology of gout. Arthritis Res. Ther..

[25] Litwin M., Saigal C. (2007). Urologic Diseases in America.

[26] Lawrence R.C., Helmick C.G., Arnett F.C., Deyo R.A., Felson D.T., Giannini E.H., Wolfe F. (1998). Estimates of the prevalence of arthritis and selected musculoskeletal disorders in the United States. Arthritis Rheum..

[27] Reichard C., Gill B.C., Sarkissian C., De S., Monga M. (2015). 100% uric acid stone formation. What makes them different?. Urology.

[28] Dumick R.N., Sandler C.M., Newhouse J.H. (1999). Nephrocalcinosis and nephrolithiasis. Textbook of Uroradiology.

[29] Curhan G.C. (2007). Epidemiology of stone disease. Urol. Clin. N. Am..

[30] Choi H.K., Mount D.B., Reginato A.M. (2005). Pathogenesis of gout. Ann. Intern. Med..

[31] Burns C.M.W.R., Longo F., Kasper D.L., Hauser S.L., Jameson J.L., Loscalzo J. (2012). Disorders of purine and pyrimidine metabolism. Harrison’s Principles of Internal Medicine.

[32] Kam M., Perl-Treves D., Caspi D., Addadi L. (1992). Antibodies against crystals. FASEB J..

[33] Kam M., Perl-Treves D., Sfez R., Addadi L. (1994). Specificity in the recognition of crystals by antibodies. J. Mol. Recognit..

[34] Kanevets U., Sharma K., Dresser K., Shi Y. (2009). A role of IgM antibodies in monosodium urate crystal formation and associated adjuvanticity. J. Immunol..

[35] Ortiz-Bravo E., Sieck M.S., Schumacher H.R. (1993). Changes in the proteins coating monosodium urate crystals during active and subsiding inflammation. Immunogold studies of synovial fluid from patients with gout and of fluid obtained using the rat subcutaneous air pouch model. Arthritis Rheum..

[36] Cherian P.V., Schumacher H.R. (1986). Immunochemical and ultrastructural characterization of serum proteins associated with monosodium urate crystals (MSU) in synovial fluid cells from patients with gout. Ultrastruct Pathol..

[37] Kozin F., McCarty D.J. (1980). Molecular orientation of immunoglobulin G adsorbed to microcrystalline monosodium urate monohydrate. J. Lab. Clin. Med..

[38] Terkeltaub R., Tenner A.J., Kozin F., Ginsberg M.H. (1983). Plasma protein binding by monosodium urate crystals. Analysis by two-dimensional gel electrophoresis. Arthritis Rheum..

[39] Busso N., So A. (2012). Microcrystals as DAMPs and their role in joint inflammation. Rheumatology.

[40] Martinon F., Pétrilli V., Mayor A., Tardivel A., Tschopp J. (2006). Gout-associated uric acid crystals activate the NALP3 inflammasome. Nature.

[41] Wallace S. (1972). The treatment of gout. Arthritis Rheum..

[42] Howell R., Seegmiller J. (1963). A mechanism of action of colchicine. Am. Rheum. Assoc..

[43] Shoji A., Yamanaka H., Kamatani N. (2004). A retrospective study of the relationship between serum urate level and recurrent attacks of gouty arthritis: Evidence for reduction of recurrent gouty arthritis with antihyperuricemic therapy. Arthritis Rheum..

[44] Seegmiller J.E. (1965). The acute attack of gouty arthritis. Arthritis Rheum. Arthritis Rheum..

[45] Wilcox W.R., Khalaf A.A. (1975). Nucleation of monosodium urate crystals. Ann. Rheum. Dis..

[46] Degan H.S., Tekgul S. (2007). Management of pediatric stone disease. Curr. Urol. Rep..

[47] Lin K.C., Lin H.Y., Chou P. (2000). The interaction between uric acid level and other risk factors on the development of gout among asymptomatic hyperuricemic men in a prospective study. J. Rheumatol..

[48] Hall A.P., Barry P.E., Dawber T.R., McNamara P.M. (1967). Epidemiology of gout and hyperuricemia. A long-term population study. Am. J. Med..

[49] Buckley H. (1951). Crystal Growth.

[50] Brazier Y. (2017). What you need to Know about Breast Augmentation?. Medical News Today.

[51] US Govt Printing Office (1992). House of Representatives FDA’s Regulation of Silicone Breast Implants: Staff Report prepared by the Human Resources and Intergovernmental Relations Subcommittee of the Committee on Government Operations.

[52] Grossman A.R. (1976). Augmentation Mammoplasty.

[53] Cronin T.D., Gerow F.J. (1964). Augmentation Mammoplasty: A New “Natural Feel” Prosthesis.

[54] International Congress Series 66. Proceedings of the Third International Congress of Plastic Surgery.

[55] Lilla J.A., Vistnes L.M. (1976). Long-term study of reactions to various silicone breast implants in rabbits. Plast. Reconstr. Surg..

[56] Wagner H., Beller F.K., Pfautsch M. (1977). Electron and light microscopy examination of capsules around breast implants. Plast. Reconstr. Surg..

[57] Peters W.J., Smith D.C. (1998). Discussion—Lipid infiltration as a possible biologic cause of silicone gel breast implant aging. Plast. Reconstr. Surg..

[58] Huang T.T., Blackwell S.L., Lewis S.R. (1978). Migration of silicone gel after the “squeeze technique” to rupture a contracted breast capsule. Case Report. Plast. Reconstr. Surg..

[59] Barker D.E., Retsky M.I., Schultz S. (1978). “Bleeding” of silicone from bag-gel breast implants, and its clinical relation to fibrous capsule reaction. Plast. Reconstr. Surg..

[60] Lane T.H., Burns S.A., Potter M., Rose N.R. (1996). Silica, silicon and silicones: Unraveling the mystery. Current Topics in Microbiology and Immunology: Immunology of Silicones.

[61] Chronology of FDA Activities Related to Breast Implants 1998. http://www.fda.gov/oca/breastimplants/bichron.html.

[62] Handel N., Jensen J.A., Black Q., Waisman J.R., Silverstein M.J. (1995). The fate of breast implants: A critical analysis of complications and outcomes. Plast. Reconstr. Surg..

[63] Ersek R.A. (1991). Rate and incidence of capsular contracture: A comparision of smooth and textured silicone double-lumen breast prosthesis. Plast. Reconstr. Surg..

[64] Terry M.B., Skovron M.L., Garbers S., Sonnenschein E., Toniolo P. (1995). Estimated frequency of cosmetic breast augmentation among US women, 1963 through 1988. Am. J. Public Health.

[65] Noone R.B. (1997). A review of the possible health implications of silicone breast implants. Cancer.

[66] Sam C.P., National Science Panel (1997). Silicone Breast Implants in Relation to Connective Tissue Diseases and Immunologic Dysfunction.

[67] Yu L.T., Latorre G., Marotta J., Batich C., Hardt N.S. (1995). In vitro measurement of silicone bleed from breast implants. Plast. Reconstr. Surg..

[68] Varaprath S. (1991). Composition analysis of mammary gel bleed. Memorandum to R.R. LeVier.

[69] Moore J.A. (1991). Final report: Identification and quantification of chemicals extractable. Internal Document to R.R. Levier.

[70] Lynch W. (1977). A current review of constrictive capsules. A Report Presented by a Consultant to Medical Engineering.

[71] Stith W.J. (1981). Letter from Medical Engineering to G.

[72] (1977). Medical Engineering Corporation, Internal Memo Dated 10/4/77.

[73] Pratt L. (1987). Competitive gel bleed tests. Medical Engineering, Internal Memorandum to Plastic Surgery Sales Force.

[74] Lykissa E.D., Kala S.V., Hurley J.B., Lebovitz R.M. (1997). Release of low molecular weight silicones and platinum from silicone breast implants. Anal. Chem..

[75] Peters W., Smith D., Lugowski S. (1996). Silicon capsule assays with low-bleed silicone gel implants. Plast. Reconstr. Surg..

[76] Schmidt G.H. (1988). Re: Cocke and Sampson: Silicone bleed associated with double-lumen breast prostheses. Ann. Plast. Surg..

[77] MSI (1991). Package Insert for Dow Corning Silastic.

[78] Peters W.J. (1991). The mechanical properties of breast prostheses. Ann. Plast. Surg..

[79] Cook R.R., Curtis J.M., Perkins L.L., Hoshaw S.J. (1998). Rupture of silicone-gel breast implants. Lancet.

[80] Collis N., Sharpe D.T. (1998). Rupture of silicone-gel breast implants. Lancet.

[81] Van Rappard J.H.A., Sonneveld G.J., van Twisk R., Borghouts J.M.H.M. (1988). Pressure resistance of breast implants as a function of implantation time. Ann. Plast. Surg..

[82] Phillips J.W., de Camara D.L., Lockwood M.D., Grebner W.C.C. (1996). Strength of silicone breast implants. Plast. Reconstr. Surg..

[83] Brandon H.J., Young V.L., Jerina K.L., Wolf C.J. Biodurability of retrieved breast implants. Proceedings of the 25th Annual Meeting of the Society for Biomaterials.

[84] Marotta J.S., Widenhouse C.W., Habal M.B., Goldberg E.P. (1999). Silicone gel breast implant failure and frequency of additional surgeries: Analysis of 35 studies reporting examination of more than 8000 explants. J. Biomed. Mater. Res..

[85] MEC (1980). Expansion of the Protocol Up-Date of 21 January 1980 for Improvement of Gel and Shell Materials. MEC Internal Memorandum.

[86] Wilfingseder P., Propst A., Mikuz G. (1974). Constrictive fibrosis following silicone implants in mammary augmentation. Chir. Plast..

[87] Luke J.L., Kalasinsky V.F., Turnicky R.P., Centeno J.A., Johnson F.B., Mullick F.G. (1997). Pathological and biophysical findings associated with silicone breast implants: A study of capsular tissues from 86 cases. Plast. Reconstr. Surg..

[88] Thomsen J.L., Christensen L., Nielsen M., Brandt B., Breiting V.B., Felby S., Nielsen E. (1990). Histologic changes and silicone concentrations in human breast tissue surrounding silicone breast prostheses. Plast. Reconstr. Surg..

[89] LeVier R.R. (1987). Medtox project. Analysis of Internal Safety Studies Relevant to Health Care Materials and Products.

[90] Smahel J. (1979). Foreign material in the capsules around breast prostheses and the cellular reaction to it. Br. J. Plast. Surg..

[91] Selmanowitz V.J., Orentreich N. (1977). Medical-grade fluid silicone. A monographic review. J. Dermatol. Surg. Oncol..

[92] Rees T.D., Ashley F.L., Delgado J.P. (1973). Silicone fluid injections for facial atrophy. A ten year study. Plast. Reconstr. Surg..

[93] Rees T.D. (1976). The current status of silicone fluid in plastic and reconstructive surgery. J. Derm. Surg..

[94] Crisp A., de Juan E., Tiedeman J. (1987). Effect of silicone oil viscosity on emulsification. Arch. Ophthalmol..

[95] Heidenkummer H.P., Kampik A., Thierfelder S. (1999). Emulsification of silicone oils with specific phases. Plast. Reconstr. Surg..

[96] Savion N., Alhalel A., Treister G., Bartov E. (1996). Role of blood components in ocular silicone oil emulsification. Studies on an in vitro model. Investig. Ophthalmol. Vis. Sei..

[97] Muruganandam L., Kunal D., Melwyn G. (2018). Studies on droplet size distribution of oil-in-water Emulsion in SMX static mixer. J. Appl. Fluid Mech..

[98] Kilpadi D.V., Feldman D.S., Lallone R. The behavior of silicone gel in saline and serum. Proceedings of the Eighth Annual Meeting of the Wound Healing Society.

[99] Yannas I.V., Burke J.F., Gordon P.L., Huang C., Rubenstein R. (1981). Design of an artificial skin. II. Control of chemical composition. J. Biomed. Mater. Res..

[100] Yannas I.V., Lee E., Orgill D.P., Skrabut E.M., Murphy S.F. (1989). Synthesis and characterization of a model extracellular matrix that induces partial regeneration of adult mammalian skin. Proc. Natl. Acad. Sci. USA.

[101] Feldman D., Hultman S., Colaizzo R., von Recum A. (1983). Electron microscopic investigation of soft tissue ingrowth into dacron velour with dogs. Biomaterials.

[102] Kossovsky N., Heggers J., Parsons R., Robson M. (1983). Analysis of the surface morphology of recovered silicone mammary prostheses. Plast. Reconstruct. Surg..

[103] Mikos A., Sarakinos G., Lyman L. (1993). Laminated three-dimensional biodegradable foams for use in tissue engineering. Biomaterials.

[104] Sanders J., Stiles C., Hayes D. (2000). Tissue response to single polymer fibers of varying diameters: Evaluation of fibrous encapsulation and macrophage density. J. Biomed. Mater. Res..

[105] Bernatchez S., Parks P., Gibbons D. (1996). Interaction of macrophages with fibrous materials in vitro. Biomaterials.

[106] Brown C.N., Finch J.G. (2010). Which mesh for hernia repair?. Ann. R. Coll. Surg. Engl..

[107] Klinge U., Klosterhalfen B., Birkenhauer V., Junge K., Conze J., Schumpelick V. (2002). Impact of polymer pore size on the interface scar formation in a rat model. J. Surg. Res..

[108] Anthony T., Bergen P.C., Kim L.T., Henderson M., Fahey T., Rege R.V., Turnage R.H. (2000). Factors affecting recurrence following incisional herniorrhaphy. World J. Surg..

[109] Annis D., Burnat A., Edwards R., Highan A., Loveday K.B., Wilson J. (1978). An elastomeric vascular prosthesis. Trans. Am. Soc. Artificial Internal. Organs..

[110] Klosterhalfen B., Junge K., Klinge U. (2005). The lightweight and large porous mesh concept for hernia repair. Expert Rev. Med. Devices..

[111] Klosterhalfen B., Hermanns B., Rosch R. (2003). Biological response to mesh. Eur. Surg..

[112] Schumpelick V., Nylus L. (2003). Meshes: Benefits and Risks.

[113] Zieren J., Neuss H., Paul M., Müller J. (2004). Introduction of polyethylene terephthalate mesh (KoSa hochfest) for abdominal hernia repair: An animal experimental study. Biomed. Mater. Eng..

[114] Boulanger G., Andujar P., Pairon J.C., Billon-Galland M.A., Dion C., Dumortier P., Brochard P., Sobaszek A., Bartsch P., Paris C. (2014). Quantification of short and long asbestos fibers to assess asbestos exposure: A review of fiber size toxicity. Environ. Health.

